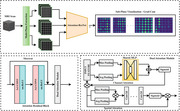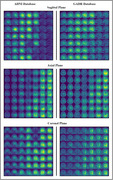# Explainable Deep Learning for Multi‐Cohort Alzheimer's Disease Classification using MRI: Insights into Pathological Brain Regions

**DOI:** 10.1002/alz70856_101859

**Published:** 2025-12-25

**Authors:** Gia Minh Hoang, Jae Gwan Kim

**Affiliations:** ^1^ Gwangju Institute of Science and Technology, Bukgu, Gwangju, Korea, Republic of (South); ^2^ Department of Biomedical Science and Engineering, Gwangju Institute of Science and Technology, Gwangju 61005, Korea, Republic of (South)

## Abstract

**Background:**

Magnetic Resonance Imaging (MRI) plays a crucial role in the early diagnosis and monitoring of Alzheimer's disease, offering detailed insights into structural and functional brain changes. However, variations in MRI data across national cohorts present significant challenges, complicating consistent and reliable diagnosis. Explainable AI with attention‐map visualization techniques, can overcome these challenges by enhancing diagnostic accuracy and enabling interpretable insights into Alzheimer's disease progression through MRI analysis. In this study, we propose a deep‐learning approach to visualize key pathological brain regions associated with AD versus Cognitive Normal (CN) classification across multi‐cohort datasets.

**Method:**

The data utilized in this study were collected from two cohorts: the Alzheimer's Disease Neuroimaging Initiative (ADNI) database (American cohort) and the Gwangju Alzheimer's and Related Dementia (GARD) database (Korean cohort). To investigate critical pathological brain regions associated with Alzheimer's disease, we trained a classification model to distinguish Alzheimer's disease (AD) from cognitively normal (CN) individuals. The model employed a ResNet‐50 backbone, integrated with an attention mechanism to enhance spatial feature extraction. To ensure the robustness of the approach, attention maps were visualized in the sagittal, axial, and coronal planes of MRI scans.

**Result:**

Our proposed method demonstrates state‐of‐the‐art performance in distinguishing AD from CN individuals, achieving 98.18% accuracy, 96.73% specificity, and 98.97% sensitivity. Attention map analysis indicates that the model primarily targets the hippocampus and temporal lobe, consistent with prior research on key pathological regions associated with Alzheimer's disease. Additionally, the model exhibits consistent performance on the GARD cohort, underscoring its generalizability and reliability.

**Conclusion:**

In summary, our method achieves outstanding performance in AD vs. CN classification, accurately identifying key pathological regions such as the hippocampus and temporal lobe. The consistent results across diverse cohorts demonstrate its robustness and potential for broader clinical implementation.